# *chaoptin*, *prominin*, *eyes shut* and *crumbs* form a genetic network controlling the apical compartment of *Drosophila* photoreceptor cells

**DOI:** 10.1242/bio.20147310

**Published:** 2014-04-04

**Authors:** Nagananda Gurudev, Michaela Yuan, Elisabeth Knust

**Affiliations:** Max Planck Institute of Molecular Cell Biology and Genetics, Pfotenhauerstrasse 108, D-01307 Dresden, Germany

**Keywords:** Microvilli, Retinal degeneration, Rhabdomere, Adhesion

## Abstract

The apical surface of epithelial cells is often highly specialised to fulfil cell type-specific functions. Many epithelial cells expand their apical surface by forming microvilli, actin-based, finger-like membrane protrusions. The apical surface of *Drosophila* photoreceptor cells (PRCs) forms tightly packed microvilli, which are organised into the photosensitive rhabdomeres. As previously shown, the GPI-anchored adhesion protein Chaoptin is required for the stability of the microvilli, whereas the transmembrane protein Crumbs is essential for proper rhabdomere morphogenesis. Here we show that *chaoptin* synergises with *crumbs* to ensure optimal rhabdomere width. In addition, reduction of *crumbs* ameliorates morphogenetic defects observed in PRCs mutant for *prominin* and *eyes shut*, known antagonists of *chaoptin*. These results suggest that these four genes provide a balance of adhesion and anti-adhesion to maintain microvilli development and maintenance. Similar to *crumbs* mutant PRCs, PRCs devoid of *prominin* or *eyes shut* undergo light-dependent retinal degeneration. Given the observation that human orthologues of *crumbs*, *prominin* and *eyes shut* result in progressive retinal degeneration and blindness, the *Drosophila* eye is ideally suited to unravel the genetic and cellular mechanisms that ensure morphogenesis of PRCs and their maintenance under light-mediated stress.

## INTRODUCTION

Photoreceptor cells (PRCs) are highly polarised cells originating from the neuroepithelium, which are characterised by an expanded apical plasma membrane, specialised to accommodate the large amount of the visual pigment rhodopsin. PRCs in the animal kingdom use two different strategies to expand their apical surface: vertebrate rods and cones, for example, expand the apical membrane by a microtubule-based modified primary cilium to form the outer segment, while many PRCs of invertebrate species form actin-based microvilli, which are organised into light harvesting compartments called rhabdomeres (Lamb, 2009; Fain et al., 2010). The microvilli of *Drosophila* rhabdomeres harbour the signalplex, a supramolecular protein complex organised by the scaffolding protein InaD, which directly binds to components of the light-dependent signalling cascade (Wang and Montell, 2007). Each rhabdomere of *Drosophila* is built from approximately 50,000 microvilli, which closely adhere to their neighbours. Each microvillus is about 1.5 µm in length and 50 nm wide. Actin filaments span the entire length of the microvilli (Arikawa et al., 1990).

The apical plasma membrane of vertebrate and *Drosophila* PRCs contains a second distinct domain, called inner segment and stalk membrane, respectively, which separates the photoreceptive outer segment/rhabdomere from the adherens junctions (AJs). Molecularly, this membrane domain is marked by the Crumbs (Crb) protein complex. The core components of this evolutionarily conserved complex are the transmembrane protein Crb, which is linked via its short cytoplasmic tail to the scaffolding proteins Stardust (Sdt)/MPP5/Pals1, *D*PATJ/PATJ and *D*Lin-7/Lin-7/Veli (Bazellieres et al., 2009; Bulgakova and Knust, 2009). In *Drosophila*, loss of any core component leads to light dependent retinal degeneration (Johnson et al., 2002; Berger et al., 2007; Bachmann et al., 2008; Chartier et al., 2012; Soukup et al., 2013). Strikingly, mutations in *CRB1*, one of the three human Crb genes, lead to blindness (den Hollander et al., 1999). This suggests that Crb proteins control similar mechanisms required to prevent PRC degeneration in vertebrates and invertebrates.

*Drosophila* PRCs develop from a simple epithelium, the eye imaginal disc. During larval development, PRCs become gradually specified and are organised into groups of eight cells, which, after recruitment of additional support cells, form the ommatidia, the units of the compound eye. At ∼37% pupal development (pd), the apical surfaces of the PRCs undergo a shift of 90°, thus adopting a lateral position, with the apical poles of the eight PRCs of an ommatidium oriented towards each other and being closely associated. At around 50% pd, the stalk membrane can be identified as a distinct portion of the apical membrane, while the microvilli of the incipient rhabdomeres increase in number and length and start to separate from those of the other rhabdomeres. At the same time, the interrhabdomeral space (IRS) is formed. This process is accompanied by a tremendous increase in the size of the PRCs, including the rhabdomere, resulting in a retinal thickness of about 100 µm (Longley and Ready, 1995).

The genetic regulation of this complex morphogenetic process has been described to some extent. The specification of the apical membrane depends on Bazooka, the *Drosophila* orthologue of Par-3, and PTEN (Pinal et al., 2006). The stalk membrane becomes visible as distinct membrane from 50% pd onwards, when Crb, which is initially spread across the entire apical plasma membrane, becomes restricted to the stalk. In the absence of Crb, the stalk membrane is reduced in length and the rhabdomeres only span the distal third of the retina (Izaddoost et al., 2002; Johnson et al., 2002; Pellikka et al., 2002). The core of the microvilli is formed by actin filaments. Actin also participates in the organisation of the rhabdomeral terminal web (RTW), a tensile sheet at the base of the rhabdomere required for microvillar actin termini linkage via Moesin. The RTW is embedded in the apical, organelle-poor cytoplasm, called ectoplasm in *Drosophila* PRCs (Xia and Ready, 2011). Moesin, the single *Drosophila* member of the ERM (ezrin–radixin–moesin) protein family, links the actin cytoskeleton to the plasma membrane. RNAi-mediated knock down of Moesin results in loosely organised microvilli, which starts being visible at around 50% pd, and strongly disorganised microvilli later on due to disrupted F-actin organisation at the rhabdomere base (Karagiosis and Ready, 2004). Microvilli formation requires actin binding proteins, such as the Wiskott–Aldrich syndrome protein WASp (Zelhof and Hardy, 2004), the actin-depolymerising factor cofilin, encoded by *Drosophila twinstar* (*tsr*) (Pham et al., 2008) or motor proteins, such as Myosin V (Li et al., 2007). The RTW not only provides a mechanical support for the microvilli but also acts as trafficking route for Rab11-dependent vesicle delivery of rhabdomeral membrane components (Satoh et al., 2005; Li et al., 2007). At around 78% of pupal development, expression of Rhodopsin 1 (Rh1), encoded by *ninaE*, is required to stabilise the RTW of PRCs R1–R6 by localising the small GTPase *D*Rac1 (Chang and Ready, 2000). In the absence of Rh1 during this period, the alignment of rhabdomeral microvilli is not maintained, resulting in their involution into the cytoplasm (Kumar and Ready, 1995; Kumar et al., 1997). However, recent results showed that *D*Rac is dispensable for this process and suggested that it may act redundantly with Cdc42 (Pinal and Pichaud, 2011).

At early pupal stages, the apical compartments of all PRCs in each ommatidium are initially attached to each other, a process mediated by the GPI (glycosylphosphatidylinositol)-anchored glycoprotein protein Chaoptin (Chp) (Krantz and Zipursky, 1990; Hirai-Fujita et al., 2008). Separation of the apical membranes requires the function of the pentaspan membrane protein Prominin (Prom) and the secreted protein Eyes shut (Eys) [also known as Spacemaker (Spam)] (Husain et al., 2006; Zelhof et al., 2006; Gurudev et al., 2013). Prom and Eys cooperatively antagonise the function of Chp in order to form an open rhabdom, in which a single, continuous IRS separates the rhabdomeres from each other. Once separated, the microvilli expand in length. Chp is further required to ensure the tight adhesion between microvilli, thus allowing the formation of a compact rhabdomere. *chp* mutant PRCs of adult flies lack fully formed microvilli (Van Vactor et al., 1988).

So far, the genetic control of rhabdomere formation by *chp*, *prom* and *eys* on the one hand and stalk membrane development, mediated by the Crb complex, on the other hand, was studied separately. Here we show that *chp* acts synergistically with *crb* to form the rhabdomere and that *crb* is part of a genetic network, which comprises *crb*, *chp*, *prom* and *eys*. Furthermore, not only *crb*, but also *prom* and *eys* are required in adult PRCs to prevent light-dependent retinal degeneration, supporting an additional functional interaction at later stages. Strikingly, all three genes are conserved, and their loss-of-function has been associated with a clinically and genetically heterogeneous group of blindness in humans. Therefore, the fly eye provides an excellent model system to further study the role of these genes during development and disease progression.

## RESULTS

### Genetic interaction between *crb* and *chp*

**Fig. 1.**
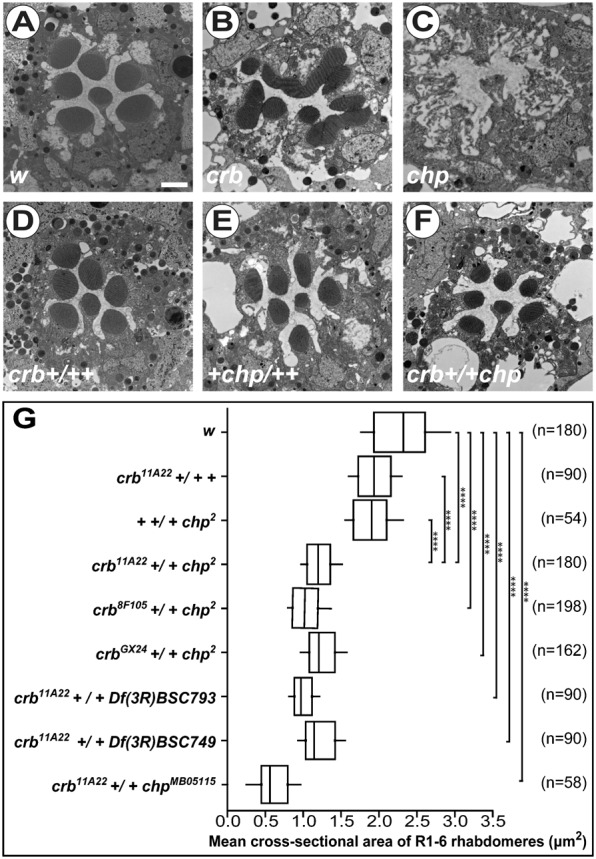
*crb* and *chp* synergistically control the width of the rhabdomere. (A–F) Electron micrographs of cross-sections of control (*w*) (A), *crb^11A22^* (B), *chp^2^* (C), *crb^11A22^* +/+ *+* (D); *+* +/+ *chp^2^* (E) and *crb^11A22^* +/+ *chp^2^* (F) adult *Drosophila* ommatidia. Scale bar: 1 µm. (G) Box-plot representing the width of rhabdomeres of indicated genotypes. Whiskers indicate 10–90% confidence interval. The width is indicated in µm^2^ and estimated by measuring cross-sectional areas of rhabdomeres from outer PRCs (R1–R6) in 1–2-day-old adult female *Drosophila* eyes. n  =  number of ommatidia.

*crb* loss of function mutations induce a pleiotropic phenotype, in which the rhabdomeres of photoreceptor cells (PRCs) are bulky, occasionally fused with each other (compare Fig. 1A to Fig. 1B), develop a shorter stalk membrane and fail to expand throughout the depth of the retina (Johnson et al., 2002; Pellikka et al., 2002). We identified *chp* in a screen performed to identify genes involved in PRC morphogenesis. For this, we screened for rhabdomere phenotypes in *crb* heterozygous flies, which were additionally heterozygous for a defined deletion on either the second (*crb^11A22^/+; Def/+*) or the third chromosome (*crb^11A22^ +/+ Def*) (to be published elsewhere). Mutations in *chp* are homozygous viable and have been shown to result in a severe reduction and disorganisation of the apical rhabdomeral microvilli in the adult eye (compare Fig. 1A to Fig. 1C) (Van Vactor et al., 1988). *chp* encodes several differentially spliced transcripts, which encode GPI-anchored adhesion molecules with 28 leucine-rich repeats (LRRs) in their extracellular domain (SMART prediction) (Reinke et al., 1988; Krantz and Zipursky, 1990). While both *crb* and *chp* mutations are fully recessive and do not show any mutant rhabdomere phenotype when heterozygous, PRCs trans-heterozygous for *crb* and *chp* develop rhabdomeres with significantly smaller width (compare Fig. 1D,E to Fig. 1F; for quantification see Fig. 1G). The length of the rhabdomeres is not affected (data not shown). The result was confirmed using various combinations of three different *crb* alleles (*crb^11A22^*, *crb^8F105^* and *crb^GX24^*) and two different *chp* alleles, *chp^2^* and *chp^MB05115^*, as well as two deficiencies removing *chp* (Fig. 1G; supplementary material Fig. S1). These results suggest that *crb* and *chp* act together to control the width of the rhabdomeres.

**Fig. 2.**
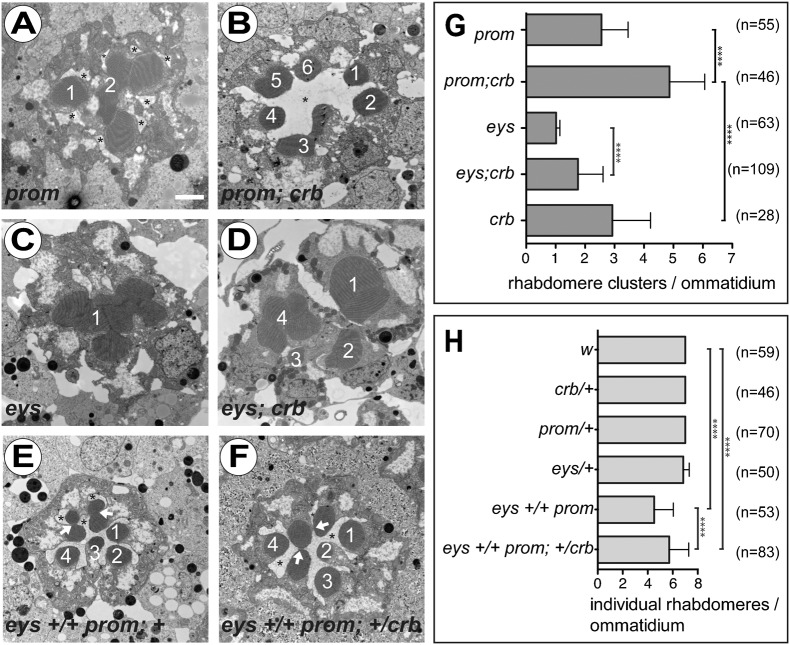
Mutation in *crb* suppresses interrhabdomere adhesion in *prom* and *eys* mutants. (A–F) Electron micrographs showing tangential sections of ommatidia of *Drosophila* with the following genotypes in *w* background: *cn bw prom^1^* (A), *cn bw prom^1^; crb^11A22^* (B), *eys^1^ cn bw* (C), *eys^1^ cn bw; crb^11A22^* (D), *+ cn bw prom^1^/eys^1^ cn bw +; +/+* (E) and *+ cn bw prom^1^/eys^1^ cn bw +; crb^11A22^*/*+* (F). Numbers in A–F depict the numbers of individual rhabdomeres or rhabdomere clusters, not the identity of PRCs. Asterisk: IRS; white arrow: rhabdomere adhesion. Scale bar: 1 µm. (G,H) Quantification of interrhabdomeral adhesion of PRCs with different genotypes. Column chart (mean ± s.d.) represents the number of single rhabdomeres or rhabdomere clusters per ommatidium (G) and average individual rhabdomeres per ommatidium (H). n  =  number of ommatidia.

The apical membranes of all PRCs of each ommatidium are initially fused to each other, and only become separated during the second half of pupal development (Longley and Ready, 1995). Data suggest that this adhesion is mediated by homophilic interaction of Chp, while the separation requires the cooperative function of Prom and Eys. Ommatidia lacking *prom* or *eys* have fused rhabdomeres (Husain et al., 2006; Zelhof et al., 2006; reviewed by Gurudev et al., 2013). Given our observation on the synergistic function of *crb* and *chp*, we anticipated a genetic interaction of *crb* with *prom* and *eys* as well. *prom* mutant ommatidia fail to separate their rhabdomeres, resulting in one or two rhabdomere clusters rather than individual rhabdomeres and an irregular IRS (Fig. 2A). Strikingly, in the absence of *crb* the *prom* mutant phenotype is largely suppressed; most of the rhabdomeres are individualised and a single, continuous IRS is formed (Fig. 2B; quantified in Fig. 2G and supplementary material Fig. S2A). In *eys* mutants, all or nearly all rhabdomeres are fused to a single rhabdomeral cluster (Fig. 2C), and no IRS is formed. In the absence of both *eys* and *crb* the number of individual rhabdomeral clusters/individual rhabdomeres is increased, but unlike in *prom;crb* double mutant PRCs no IRS is formed in *eys;crb* double mutants (Fig. 2D; quantified in Fig. 2G and supplementary material Fig. S2A). Concomitant removal of one copy of *prom* and *eys* results in partial fusion of rhabdomeres (Fig. 2E, white arrows) (Zelhof et al., 2006), which is variable along the length of the retina. The fusion phenotype is rescued along the entire length of the rhabdomeres by removing just one copy of *crb* (Fig. 2F; quantified in Fig. 2H, supplementary material Fig. S2B and data not shown). Based on these results we suggest that loss of *crb* reduces the adhesive activity of Chp between individual rhabdomeres.

### Crb and Chp affect localisation of each other in adult PRCs

**Fig. 3.**
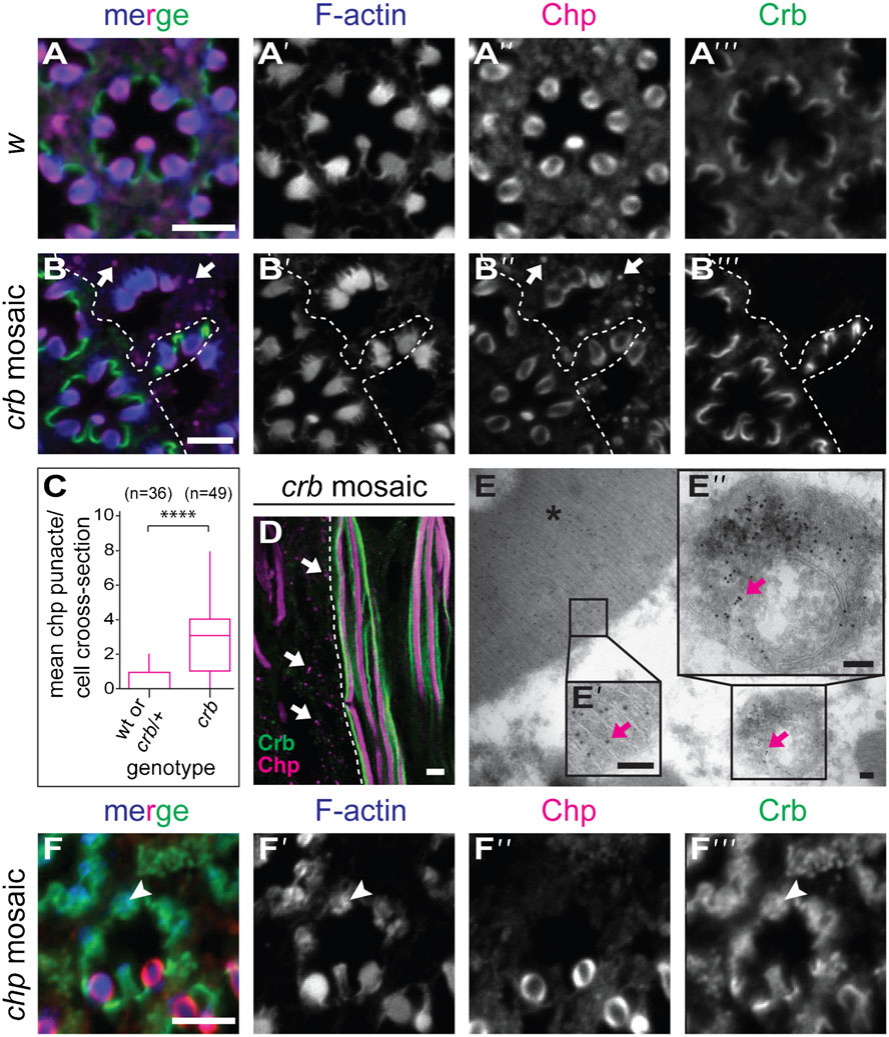
Localisation of Crb and Chp in wild-type and mutant PRCs. (A,B,D) *Drosophila* adult ommatidia. Genotypes are *w* (A), *crb^11A22^ mosaic* (B,D,E) and *chp^2^ mosaic* (F). Confocal images of immunostainings on tangential (A,B,F) and longitudinal (D) sections of PRCs stained for F-actin (blue), Chp (magenta) and Crb (green). White arrows in panels B,B″ and D point to intracellular Chp punctae in *crb* mutant cells. (C) Box-plot representing the number of cytosolic Chp positive punctae per cell per cross-section of wt, *crb*/+ and *crb*/*crb* mutant PRCs. Whiskers indicate 5–95% confidence interval. Statistical significance is analysed with Kruskal–Wallis test, followed by Dunn's multiple comparisons test. ****p<0.0001. (E–E″) Immunoelectron micrograph showing the localization of Chp (10 nm gold particle) (magenta arrows) in a cross-section of *crb^11A22^* mutant PRC. Chp is localized in the rhabdomere (E,E′, asterisk) and in a multivesicular body (E″, arrow). (F) Confocal images of immunostainings on tangential sections of PRCs stained for F-actin (blue), Chp (magenta) and Crb (green). White arrowhead points to the apical membrane in *chp* mutant cells, which is no longer subdivided into rhabdomere and stalk. Scale bars: 5 µm (A,B,D,F), 100 nm (E).

The genetic interactions shown above suggest that Crb regulates Chp. Both in wild-type (Van Vactor et al., 1988; Zelhof et al., 2006; Kanie et al., 2009; Sanxaridis and Tsunoda, 2010; Rosenbaum et al., 2012; Yano et al., 2012) and in *crb* heterozygous PRCs, Chp is strongly enriched in the rhabdomere (Fig. 3A,A″ and data not shown). In *crb* mutant PRCs, identified by the absence of Crb protein from the stalk membrane, Chp is still highly enriched in rhabdomeres, but it is also detected in intracellular dot-like structures (Fig. 3B,B″,D, white arrows; quantification in Fig. 3C). This intracellular localisation was confirmed by immuno-EM analysis (Fig. 3E–E″, magenta arrow). Whether these intracellular sites represent compartments of the degradation pathway, such as multivesicular bodies (Sapp et al., 1991) as suggested by Fig. 3E″, needs further analysis. These data suggest that Crb ensures the proper localisation of Chp in adult PRCs.

To analyse the effect of *chp* on Crb, we made use of the allele *chp^2^*, which has been described as a functional null allele carrying a deletion that removes the 3′ 2785 nucleotides of the gene (Van Vactor et al., 1988). We confirmed that the deletion starts 3′ to exon 6 and removes the remaining coding region. The exact localisation of the second breakpoint could not be determined (supplementary material Fig. S3B). Other *chp* alleles were also analysed, including several published (Sanxaridis and Tsunoda, 2010) and newly induced alleles (see Materials and Methods), following determination of their molecular lesion (summarised in supplementary material Table S1 and Fig. S3). All null alleles gave rise to the same mutant phenotype (Fig. 3F; supplementary material Fig. S4). In wild-type PRCs, Crb is restricted to the stalk membrane (Fig. 3A,A′″). In all *chp* alleles tested and in *chp*-RNAi, Crb protein is still associated with the apical membrane of PRCs (Fig. 3F,F′″, arrowhead; supplementary material Fig. S5). However, Crb as well as actin cover the entire apical surface in PRCs of amorphic allele *chp^2^* (Fig. 3F–F′″, white arrowhead), indicating that subdivision of the apical membrane into a defined stalk membrane and the rhabdomere is abolished. Nevertheless, the rudimentary rhabdomere spans the depth of the retina (supplementary material Fig. S5). From these results we conclude, that *crb* is required for optimal localisation of Chp at the apical membrane. *chp* is essential for microvilli stability, a prerequisite for the proper subdivision of the apical membrane into stalk and rhabdomere in adult PRCs.

### The observed *chp* mutant phenotype starts at midpupal development

**Fig. 4.**
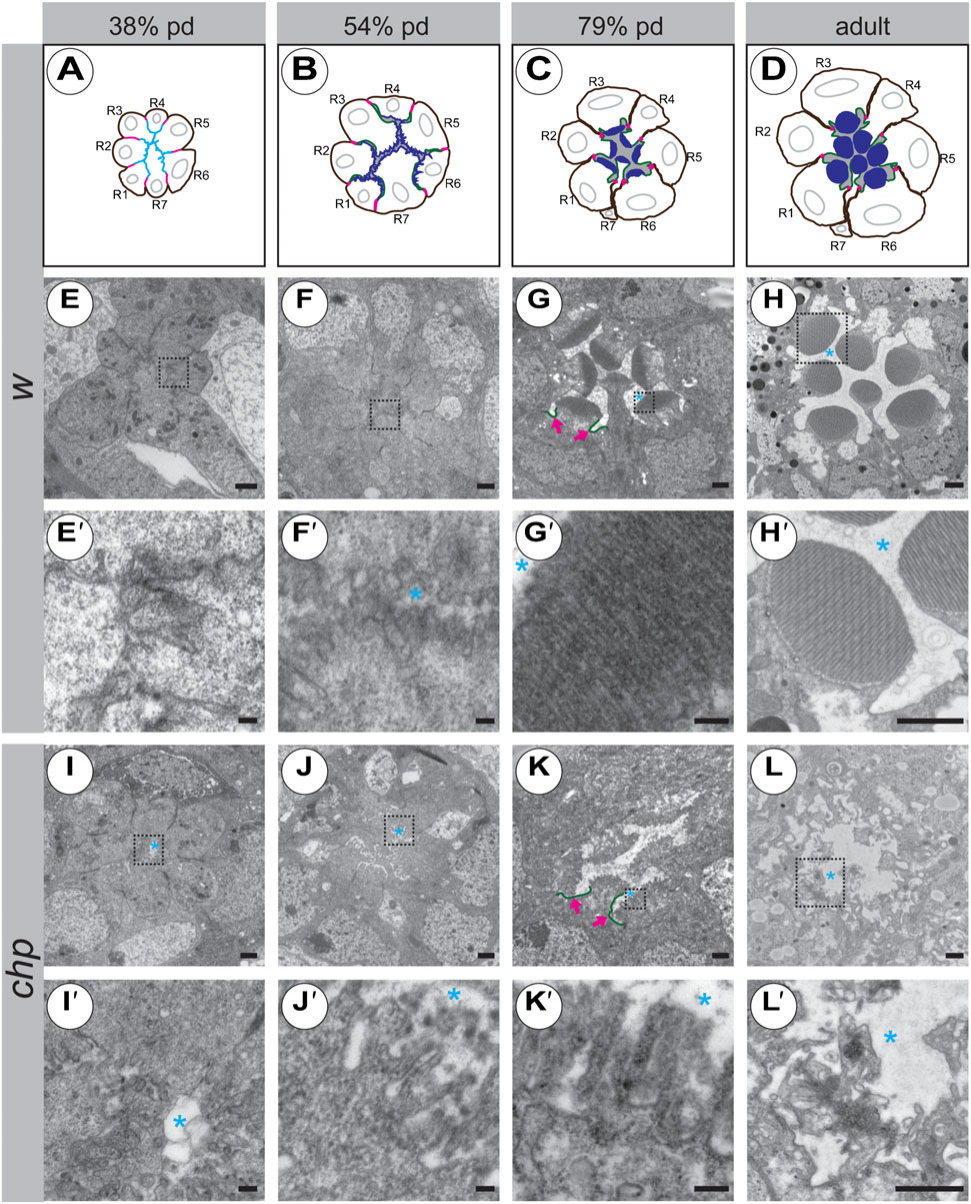
Development of the apical compartment in *chp* mutant PRCs. (A–D) Cartoons of different developmental stages in wild-type ommatidia (not taking into account the correct contacts made by individual rhabdomeres). Magenta: adherens junction. Cyan: common apical surface in panel A, as deduced by co-localisation of Crb and F-actin. Green: Crb, highlighting the stalk membrane. Blue: F-actin, highlighting the microvilli. Grey: interrhabdomerel space. R1–R7  =  number of PRCs. pd  =  pupal development. (E–L′): Electron micrographs of tangential sections of wild-type (E–H′) and *chp^2^* mutant (I–L′) ommatidia at 38% pd (E,E′,I,I′), 54% pd (F,F′,J,J′), 79% pd (G,G′,K,K′) and in the adult (H,H′,L,L′). The first defects in *chp* mutant ommatidia can already be detected at 38% pd, in that the microvilli are not as closely associated with each other as in wild type (compare panel E′ to panel I′). Scale bars: 1 µm (E–H,H′,I–L,L′) and 100 nm (E′–G′,I′–K′); blue asterisk, IRS; magenta arrows, stalk membrane (labelled in green).

The adult phenotype of PRCs mutant for a loss-of-function *chp* allele is characterised by a severe reduction in the number and length of microvilli (Fig. 1C) and defective differentiation of the apical compartment (Fig. 3F; supplementary material Fig. S4). To determine the time point at which development of the microvilli starts to fail in the mutant, we compared PRCs of wild-type and *chp* mutant flies at different stages of pupal development. Shortly after puparium formation the apical surfaces of wild-type PRCs are tightly associated with each other (Longley and Ready, 1995) (Fig. 4A,E,E′). In *chp* mutants at a comparable stage, a small space can often be observed, suggesting that the apical membranes of opposing PRCs are less closely associated with each other (Fig. 4I,I′, blue asterisk). The difference between wild type and mutant becomes more pronounced at 54% pd. In wild type, PRCs at this stage are arranged in a stereotypic way, with their apical surface still in close association with that of their neighbours (Fig. 4B,F,F′). Thereby, the apical surface contacts either an incipient rhabdomere or a neighbouring stalk membrane, which is now clearly distinguishable from the incipient rhabdomere. The length of the microvilli is rather uniform (Fig. 4F′). In contrast, the microvilli of *chp* mutant PRCs are disorganised and more sparse than those in wild type (Fig. 4J,J′). Very often, they are pointing towards a central cavity, rather than being close to a neighbouring rhabdomere or stalk membrane. Wild-type PRCs at 79% pd exhibit clearly formed rhabdomeres, embedded in a now distinct IRS. They have tightly packed and properly aligned microvilli, which touch neighbouring PRCs only occasionally. The stalk membrane can be clearly distinguished (Fig. 4G,G′, highlighted in green in Fig. 4C). In *chp* mutant PRCs of this stage, the apical microvilli are less densely packed and rather stand out as individual microvilli. The stalk is visible as a smooth membrane (Fig. 4K,K′, indicted by magenta arrows). The rhabdomeres of adult wild-type flies show tightly packed microvilli and are well separated from each other by the IRS (Fig. 4D,H,H′). In contrast, microvilli of adult *chp* mutant PRCs deteriorate, with individual microvilli hard to distinguish (Fig. 4L,L′), hence termed “rudimentary rhabdomeres” (Van Vactor et al., 1988). Taken together, *chp* controls two distinct adhesion processes in developing PRCs: it ensures adhesion of the apical membranes of opposing PRCs at early stages, and elongation and tight packing of microvilli within the rhabdomeres at later stages.

### *prom* and *eys*, but not *chp* mutant PRCs degenerate

**Fig. 5.**
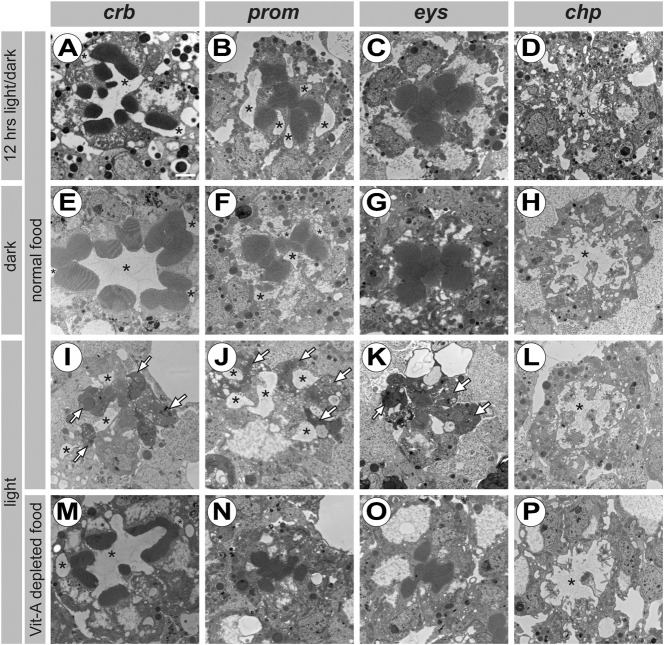
Effect of constant light exposure on the survival of *crb*, *prom*, *eys* and *chp* mutant PRCs. Electron micrographs of ommatidial cross-sections of *Drosophila* raised on standard (A–L) or vitamin A-depleted *Drosophila* medium (M–P) and kept under various light conditions. Genotypes are: *crb^11A22^* (A,E,I,M), *cn^1^ bw^1^ prom^1^* (B,F,J,N), *eys^1^ cn^1^ bw^1^* (C,G,K,O) and *chp^2^* (D,H,L,P), all in a *w* genetic background. *crb*, *prom* and *eys* mutant PRCs do not show any signs of retinal degeneration when kept under a 12 hrs light/dark cycle (A–C) or in constant darkness (E–G). When kept in constant light, they display characteristics of degeneration (I–K), such as condensed cytoplasm (white arrows) and missing rhabdomeres. Degeneration under constant illumination is prevented when animals were raised in a vitamin A-depleted medium (M–O). Under all tested conditions, *chp* mutant PRCs do not show any major signs of retinal degeneration (D,H,L,P). Scale bar: 1 µm; asterisk: IRS.

Given the close genetic interaction between *crb*, *chp*, *prom* and *eys*, and the observation that *crb* mutant PRCs undergo light-dependent degeneration (Johnson et al., 2002; Chartier et al., 2012), we analysed the effect of constant light exposure on the survival of PRCs mutant for *chp*, *prom* and *eys*. When kept in constant darkness or in a 12 hrs light/dark cycle, none of the mutant PRCs degenerated (Fig. 5A–H). However, when kept under constant illumination for 5 days, PRCs mutant for either *prom* or *eys* undergo light-dependent degeneration similar as *crb* mutant PRCs. The majority of PRCs show typical signs of degeneration, such as condensed cytoplasm (Fig. 5I–K, white arrows). Although the phenotype of *chp* mutants was previously interpreted as degeneration (Van Vactor et al., 1988; Rosenbaum et al., 2012), detailed inspection reveals that *chp* mutant PRCs do not degenerate when kept under any of these light conditions (Fig. 5D,H,L). Despite a nearly complete loss of microvilli, we could not detect any sign of apoptosis, such as cytoplasmic condensation, which is clearly visible in degenerating PRCs of the other genotypes (compare Fig. 5I–K to Fig. 1L).

We previously showed that degeneration of *crb* mutant PRCs can be prevented when larvae were raised in vitamin A-depleted food (Fig. 5M) (Johnson et al., 2002), which reduces the amount of Rh1 synthesis to about 3% of its normal amount in wild type. Strikingly, retinal degeneration of *prom* and *eys* mutant PRCs could also be prevented in the absence of dietary vitamin A (Fig. 5N,O). The phenotype of *chp* mutant PRCs was unaltered under light stress, with or without dietary vitamin A (Fig. 5D,H,L,P), which could be explained by a strongly reduced level of Rh1 in *chp* mutant PRCs (Rosenbaum et al., 2012). These results suggest that *crb*, *prom* and *eys* prevent light-dependent degeneration of PRCs by a similar cellular mechanism.

## DISCUSSION

Two genetic systems have been described previously to control rhabdomere and stalk membrane development, *chp*, *prom* and *eys* on the one hand, and *crb* on the other hand. Here we show that the two systems form a common genetic network to assure the development of the highly elaborated apical surface of *Drosophila* PRCs. In addition, *crb*, *prom* and *eys* prevent light-dependent retinal degeneration, which can be prevented by dramatically reducing Rh1 levels. This suggests that these three genes are also functionally linked in adult PRC homeostasis.

The GPI-anchored protein Chp is required for three distinct processes during development of *Drosophila* PRCs. First, it ensures interrhabdomeral adhesion at early stages of pupal development. A failure to perform this function becomes obvious already at 38% pd in *chp* mutant PRCs, when the developing apical protrusions are less closely interdigitated with those of the neighbouring cells. Most insects, such as the honeybee *Apis mellifera*, the mosquito *Anopheles gambiae* or the flour beetle *Tribolium castaneum*, form a fused rhabdom without an IRS, in which all rhabdomeres of an ommatidium are closely attached to each other, even in the adult fly. In comparison to a closed rhabdome, the open rhabdome found in most flies, including *Drosophila*, confers increased sensitivity and a better signal-to-noise ratio (reviewed by Osorio, 2007). Interestingly, although the *eys* homologue exists in these species, it is not expressed in the visual system of pupae, whereas the *chp* and *prom* homologues are (Husain et al., 2006; Zelhof et al., 2006).

The second function of Chp is required later for the close alignment of neighbouring microvilli within a rhabdomere. Homophilic adhesion has been described for other systems to stabilise microvilli. In the cochlea of mammals, for example, protocadherin 15 (PCDH15) provides lateral links to connect the stereocilia, actin-based microvilli located on the apical surface of auditory hair cells, which are specialised to receive sensory input by sound. Mutations in *PCDH15* are associated with Usher syndrome type 1F, a recessive disease characterised by retinopathy and hearing loss (Ahmed et al., 2001; Alagramam et al., 2001; El-Amraoui and Petit, 2005). Strikingly, the *Drosophila* homolog of PCDH15, Cad99C, is required for microvilli stability in the developing egg chamber (Schlichting et al., 2006).

Our data reveal that the phenotype of *chp* adult eyes is much stronger than that observed at 79% pd, suggesting that Chp is particularly important during the final stages of pupal development. This could be explained either by assuming that the increased length of microvilli require stronger adhesion for their stabilisation. Alternatively, other proteins, such as Rh1 or Php13, may contribute to stabilise microvilli at the end of pupal development. Rh1 plays an essential role in rhabdomere morphogenesis and is synthesised from 78% pd onwards (Kumar and Ready, 1995). In fact, Rh1 levels are strongly reduced in freshly eclosed *chp^2^* adult flies (Rosenbaum et al., 2012). However, in contrast to mutations in *ninaE*, which encodes Rh1, microvilli do not protrude into the cytoplasm in *chp* mutant PRCs, suggesting that the reduced amount of Rh1 is sufficient to stabilise the RTW. PRCs lacking the transcription factor Php13 show a similar phenotype as *chp* mutant PRCs until 60% pd, which becomes more severe after 72% pd (Zelhof et al., 2003). Together with *orthodenticle* (*otd*)/*ocelliless* (*oc*), *Pph13* regulates the expression of rhodopsin and *chp*, and probably other, not yet identified target genes required for microvillar morphogenesis (Mishra et al., 2010).

The third function of Chp is required, directly or indirectly, to subdivide the apical membrane into rhabdomere and stalk. Adult *chp* mutant PRCs have only few and strongly disorganised microvilli and no distinguishable stalk membrane, resulting in the spreading of the stalk membrane specific protein Crb throughout the apical surface.

Our data reveal an unexpected role of *crb* for the localisation of Chp in adult PRCs. The accumulation of Chp in multivesicular bodies in *crb* mutant PRCs suggests that *crb* is required for efficient localisation of Chp in the rhabdomeral membrane. We previously showed that in the absence of *crb* Rh1 accumulates in intracellular dots of unknown identity in adult PRCs (Pocha et al., 2011). Recently, an RNAi screen aimed to identify regulators of polarity proteins in PRCs was published. This screen identified several genes, the knock-down of which resulted in accumulation of the apical marker Chp in intracellular dots. The genes included regulators of protein/vesicle transport, such as Sec10, RabX4 and transportin, but also genes predicted to be involved in protein degradation (Yano et al., 2012). In addition, regulators of protein synthesis and modification are required to ensure proper delivery of Chp to and stabilisation at the apical surface (Rosenbaum et al., 2012). Whether a direct interaction between Crb and Chp occurs at any of these steps, or whether Crb is indirectly involved in Chp trafficking and stability is not known. It is tempting to speculate that the same relationship between Crb and Chp functions exists already at early stages of pupal development, when Prom and Eys antagonise Chp function (Zelhof et al., 2006). Loss of *crb* rescues the interrhabdomeral adhesion of *prom* and *eys* mutant PRCs, but whether it acts directly on any of these genes/proteins or in an indirect way has to be determined.

Beside their role in antagonising Chp function in the first half of pupal development, we present data showing that both Prom and Eys are additionally required for the survival of PRCs under light stress, a function also attributed to *crb* (Johnson et al., 2002). Several mechanisms are discussed, which prevent retinal degeneration in flies (Colley, 2012; Nie et al., 2012; Raghu et al., 2012). Accumulation of intracellular Rh1 has been suggested to trigger light dependent PRC degeneration in *crb* mutant PRCs (Pocha et al., 2011; Hollingsworth and Gross, 2012). It can be prevented by reduction of dietary vitamin A (Johnson et al., 2002), which has been shown to lower the amount of Rh1 to about 3% (Nichols and Pak, 1985). Raising *prom* and *eys* mutant animals in vitamin A depleted food also prevented light-dependent retinal degeneration, suggesting that *prom* and *eys* contribute to cell survival under light stress, directly or indirectly, by regulating Rh1. Interestingly, mutations in the human orthologues Prominin 1 (*PROM1*) and *EYS* are associated with autosomal-recessive retinitis pigmentosa and macular degeneration. Similar as in flies, mutations in mouse *Prom1* result in defects in PRC morphogenesis, followed by degeneration (Maw et al., 2000; Zhang et al., 2007; Abd El-Aziz et al., 2008; Collin et al., 2008; Yang et al., 2008; Zacchigna et al., 2009). Considering that the expansion and organisation of the apical surface occurs by different mechanisms in vertebrates and *Drosophila* – formation of membrane discs vs microvilli – these proteins seem to control a very basic, evolutionarily conserved cell biological function, required for the maintenance of apical, light-sensing membrane integrity. This assumption is further strengthened by recent results showing that PROM1 can substitute the *Drosophila* protein in *prom* mutant fly PRCs and that expression of *PROM1*, which carries a mutation that has been associated with the development of blindness (hProm1R373C), results in morphologically defective rhabdomeres when expressed in fly PRCs (Nie et al., 2012).

In contrast to *prom*, *eys* and *crb*, PRCs mutant for *chp* do not degenerate, even when exposed to constant light. This is in contrast to previously published papers, which have interpreted the lack of rhabdomeral microvilli as indication of apoptosis (Van Vactor et al., 1988; Rosenbaum et al., 2012). However, in histological sections of *chp* mutant ommatidia we always detected seven cell bodies with rudimentary rhabdomeres, even after several days of light exposure. *chp* mutant PRCs have low levels of Rh1 (Rosenbaum et al., 2012), which is probably not sufficient to accumulate in toxic doses even under light stress and therefore no degeneration occurs.

Taken together, our work has unravelled genetic interactions between *crb*, *chp*, *prom* and *eys*, which build an important genetic network to ensure proper development of microvilli and formation of an open rhabdom in *Drosophila* ommatidia. Given the observation that all four proteins are localised apically, it is possible that they build an apical regulatory network to perform this function, but the molecular details of this interaction are not known. In addition, *crb*, *prom* and *eys* are required in adult eyes for PRC survival upon light stress, a function that is conserved during evolution. This makes the *Drosophila* eyes an ideal model to understand the corresponding processes in humans, which, when perturbed, often result in diseases such as retinitis pigmentosa or microvillus inclusion disease (Ameen and Salas, 2000; Bazellieres et al., 2009; Bulgakova and Knust, 2009; Gurudev et al., 2013).

## MATERIALS AND METHODS

### *Drosophila* stocks/experimental genotypes

*Drosophila* lines were maintained at 25°C on standard *Drosophila* food unless mentioned otherwise. The following *Drosophila* stocks were used: *w^1118^*, *Oregon-R* or *w^1118^*; *cn^1^ bw^1^* as wild type. Loss-of-function alleles: *crb^11A22^* and *crb^8F105^* (Jürgens et al., 1984; Tepass and Knust, 1993; Wodarz et al., 1993), *crb^GX24^* (Huang et al., 2009), *chp^2^* (Van Vactor et al., 1988); *chp^Z3513^*, *chp^Z5240^* and *chp^Z4345^* (Zuker collection) (Koundakjian et al., 2004; Sanxaridis and Tsunoda, 2010), a kind gift from Tsunoda Lab; *chp^MB05115^* and *Df(3R)BSC793* and *Df(3R)BSC749* (Bloomington); *chp^SS52^* (this study), *prom^1^* and *eys^1^* (*spam^1^*) (Zelhof et al., 2006), a kind gift from C. Zuker, *eyFLP; Rh1-GAL4; FRT82B w^+^ Bcl3R3/MKRS, eyFLP; Rh1-GAL4; FRT82B w^+^/MKRS, eyFLP; Rh1-GAL4; FRT82Bcrb^11A22^/TM6B* (Richard et al., 2009). RNAi stocks V105053 and V39177 from Vienna Drosophila Research Centre (VDRC) (Dietzl et al., 2007). *Drosophila* manipulations were done in accordance with standard techniques.

### Generating the hypomorphic allele *chp^SS52^*

*chp^MB05115^* is a mutation induced by the insertion of a *minos*-element in the first intron (Flybase). Homozygous *w; Mi{ET1}chp^MB05115^* flies (Bloomington stock no. 24321), carrying a *Minos*-transposon inserted in the *chp* locus (FlyBase) (supplementary material Fig. S3) were crossed with *w^1118^*; *sna^Sco^/SM6a, P{w[+mC] = hsILMiT}2.4* flies, which carry a heat-shock inducible Minos-transposase (Bloomington stock no. 24613). The following steps were performed as described previously (Metaxakis et al., 2005). Homozygous stocks obtained after mobilisation of the Minos element were screened for defects in the rhabdomere using the optical neutralisation assay (see below). One stock, *chp^SS52^*, showed mild rhabdomere defects severely affecting R7. The hypomorphic phenotype was confirmed by electron microscopic analysis of *chp^SS52^/Df(3R)BSC793* adult ommatidia.

### Optical neutralization assay

The technique was performed as described previously with modifications (Franceschini, 1972; Morante and Desplan, 2011). In brief, dissected *Drosophila* heads were mounted with immersion oil on a bridged glass slide to preserve the three-dimensional structure and covered with a glass slide. Bright field images were taken using an oil immersion objective lens 63×, Zeiss AxioImager.Z1.

### Electron microscopy

Retinas from 1–2-day-old adult female flies were fixed, sectioned and photographed as described previously (Richard et al., 2006; Mishra and Knust, 2013) with minor modifications. Adult fly heads were bisected along the midline, and fixed for first 20 min in 25% glutaraldehyde in PB (0.1 M phosphate buffer [pH 7.2]), followed by fixation in 1% osmium tetroxide + 2% glutaraldehyde for 30 min at 4°C and followed by 2% osmium tetroxide for 30 min at 4°C. After dehydration with ethanol, eyes were infiltrated and embedded in Durcupan and semi- (2 µm) and ultra- (70 nm) thin sections were cut using a Leica Ultracut UCT. Semi-thin sections were stained with toluidine blue and imaged using Zeiss AxioImager.Z1. Ultra-thin sections were contrasted with 2% uranyl acetate in pure water for 10 min and lead citrate for 5 mins, and analyzed using a Morgagni electron microscope (FEI Company, 80 kV) and distal eye sections were imaged using Morada digital camera (SIS). Pupae were staged (Walther and Pichaud, 2006), fixed, sectioned, contrasted and imaged essentially as described previously (Longley and Ready, 1995), except that the retinal–brain complex from staged pupae was dissected and fixed on ice.

### Quantification of rhabdomere size

All measurements were obtained from electron micrographs taken from cross-sections from the distal third of compound eyes of 1–2-day-old adult female *Drosophila*. The cross sectional area of R1–R6 rhabdomeres was measured from at least 3 different individual flies per genotype using Fiji software. Graphs were drawn and statistical analyses were performed using Prism software.

### Immuno-electron microscopy

Heads from 1–2-day-old adult female flies were bisected and fixed in 4% formaldehyde in PB for 30 min at RT (room temperature) and 12 hrs at 4°C on a rotator. The samples were washed 3 times with PB and cryoprotected in 2.7 M sucrose in PB for 12 hrs at 4°C. Each eye was picked up with a pin and quickly frozen in liquid nitrogen. Ultrathin sections (70 nm) were cut using a Leica EM UC6, washed 2×10 min with PBG (PB with 0.5% BSA and 0.2% Gelatin), followed by incubation with MAb24B10 (1:20 in PBG) for 1 hr. Specimens were washed 6×5 min with PBG and incubated with secondary goat anti-mouse IgG coupled to 10 nm gold particle for 1 hr, followed by 6×5 min washes with PBG and 6×2 min washes with PB. After post-fixation with 1% glutaraldehyde for 5 min, specimens were washed 2×2 min with PB, 6×2 min with pure H_2_0 and stained with 2% uranyl acetate. The grid was coated in 0.1% methylcellulose + 2% uranyl acetate. Grids with ultrathin sections were imaged using a Morgagni (FEI company, 80 kV) electron microscope and micrographs were taken with Morada camera and ITEH software (Olympus).

### Light induced degeneration assay

For light induced degeneration assay, 1–2-day-old adult flies raised in 12-hr light/dark cycles at 25°C were exposed to constant light (1.700±20 lux) for 5 days at 25°C and 60% humidity. The carotenoid-free medium was prepared as previously described (Pocha et al., 2011).

### Immunohistochemistry

1–2-day-old adult female *Drosophilae* were fixed, sectioned, stained and mounted as previously described (Muschalik and Knust, 2011). In brief, adult flies were fixed with Stefanini's fixative (8% PFA, 15% picric acid, and 75 mM Pipes; pH 7.4) for 60 min at RT, washed 3× with PBS (phosphate-buffered saline), pH 7.2. Heads were dissected from the body and cryopreserved by incubation in 10% sucrose in PBS, pH 7.2, for 30 min at RT and then in 25% sucrose in PBS, pH 7.2, overnight at 4°C. Heads were then embedded in Richard-Allan Scientific Neg-50 molds (Thermo Fisher Scientific), deep frozen on dry ice and stored at −80°C until used. 12-µm thick cryo-sections were cut on a cryostat microtome (HM560; Thermo Fisher Scientific). The cryo-sections were collected on coated glass slides (Marienfeld), surrounded with a layer of hydrophobic compound (ImmedgePEN, Vector), permeabilised for 1 hr in PBT [PBS with 0.1% Triton X-100, pH 7.2] and incubated in blocking buffer [PBS with 4% BSA (bovine serum albumin)] 2 hrs at room temperature, followed by incubation over night at 4°C with the primary antibody in blocking buffer. The primary antibody was removed and sections were washed for 3×20 min in blocking buffer before incubation with the secondary antibody and Alexa-Fluor-phalloidin for 2 hrs at RT. After washing (3×20 min) in blocking buffer, sections were mounted in Mowiol (Calbiochem)-containing 4% DABCO (Sigma). The following antibodies were used: rat anti-Crb2.8 antibody (1:1000) (Richard et al., 2006); mouse anti-Chp (24B10, 1:200, DSHB). Alexa-Fluor-647, Alexa-Fluor-555, Alexa-Fluor-488 (Invitrogen) were used as secondary antibodies at 1:400 dilution. Rhabdomeres were visualized by labelling F-actin with Alexa-Fluor-488-phalloidin at 1:40 (Invitrogen). Images were taken either on Zeiss LSM 510/710 confocal microscopes.

### Production of anti-Chp N7A antibody

N7A is a polyclonal rabbit ant-Chp antibody, which was generated by immunizing rabbits with two synthetic peptides from the N-terminus [KLDLSGDRNDPTNLQT] of Chp-PA, PF and PD, and C-terminus [YNSSWSGRNEHGGMYH] of Chp-PA, PE, PF and PD (Speedy-28 day package, Eurogenetec Deutschland GmbH, Köln, Germany). The specificity of N7A antibody was confirmed by western blots (1:2000) using extracts from homogenized wild-type and *chp* mutant fly heads.

### Image processing

Images were processed using Fiji and/or Adobe Photoshop-CS5. Image manipulation was fully compliant with the image guidelines for proper digital image handling outlined in Rossner and Yamada (Rossner and Yamada, 2004).

## Supplementary Material

Supplementary Material
