# Supplementary material for: chaoptin, prominin, eyes shut and crumbs form a genetic network controlling the apical compartment of Drosophila photoreceptor cells
Source: Biol Open. 2014 Apr 4;3(5):332–41. doi: 10.1242/bio.20147310 (PMC4021355; doi:10.1242/bio.20147310)
Supplement: Supplementary Material [file supp_3_5_332__index.html]

chaoptin, prominin, eyes shut and crumbs form a genetic network controlling the apical compartment of Drosophila photoreceptor cells — chaoptin, prominin, eyes shut and crumbs form a genetic network controlling the apical compartment of Drosophila photoreceptor cells — Supplementary Material 

# *chaoptin*, *prominin*, *eyes shut* and *crumbs* form a genetic network controlling the apical compartment of *Drosophila* photoreceptor cells

## bio.20147310 Supplementary Material

**Files in this Data Supplement:**

- Supplementary Material - Nagananda Gurudev et al. doi: 10.1242/bio.20147310
