# Supplementary material for: chaoptin, prominin, eyes shut and crumbs form a genetic network controlling the apical compartment of Drosophila photoreceptor cells
Source: Biol Open. 2014 Apr 4;3(5):332–41. doi: 10.1242/bio.20147310 (PMC4021355; doi:10.1242/bio.20147310)
Supplement: Supplementary Material [file supp_bio.20147310_bio.20147310-s1.pdf]

Biology Open (2014) 000, 1–10 doi:10.1242/bio.20147310

Supplementary Material

Nagananda Gurudev et al. **doi: 10.1242/bio.20147310**

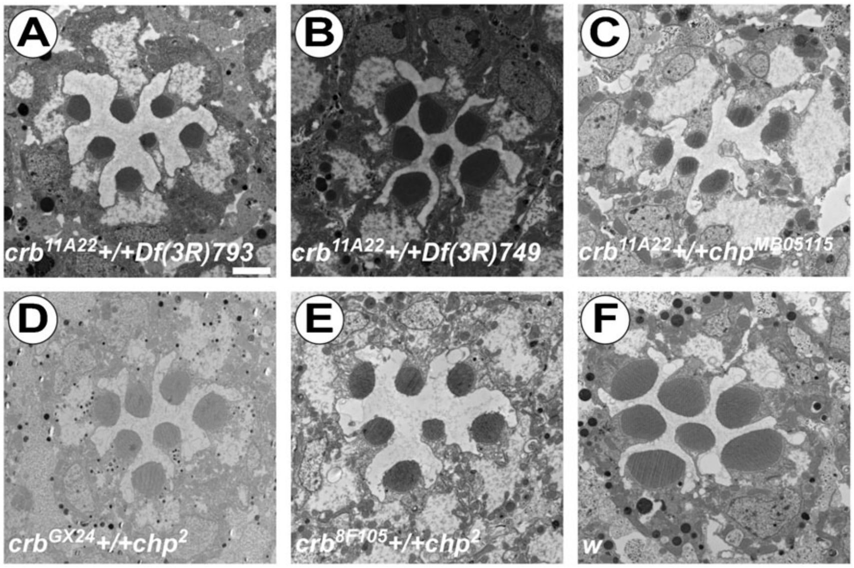

Fig. S1. Genetic interactions between different *crb* and *chp* mutant alleles. Electron micrographs of cross-sections of *w; crb^{11A22} +/+ Df(3R)BSC793* (A), *w; crb^{11A22} +/+ Df(3R)BSC749* (B), *w; crb^{11A22} +/+ chp^{MB05115}* (C) *w; crb^{GX24} +/+ chp^2* (D), *w; crb^{8F105} +/+ chp^2* (E) and *w* (F) adult ommatidia. Scale bar: 1 μm.

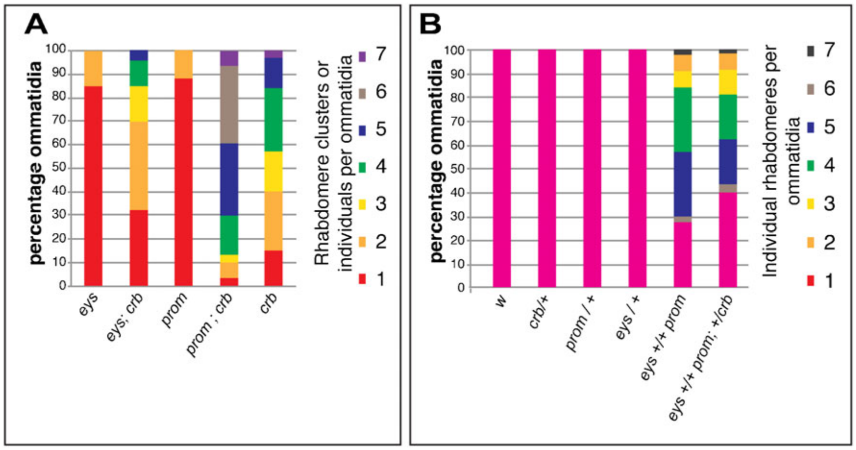

Fig. S2. Quantification of interrhabdomeral adhesion in different mutant backgrounds. Graphs showing quantification of individual rhabdomeres or rhabdomere clusters per ommatidium in *w; eys cn bw*, *w; eys cn bw; crb^{11A22}*, *w; cn bw prom*, *w; cn bw prom;crb^{11A22}*, *crb^{11A22}* (A) and *w*, *w; eys cn bw/+*, *w; cn bw prom/+*, *w*; +; *crb^{11A22}/+*, *w; eys cn bw +/+ cn bw prom*, *w; eys cn bw +/+ cn bw prom; crb^{11A22}/+* (B). n = number of ommatidia, $n>30$ (A); $n>50$ (B).

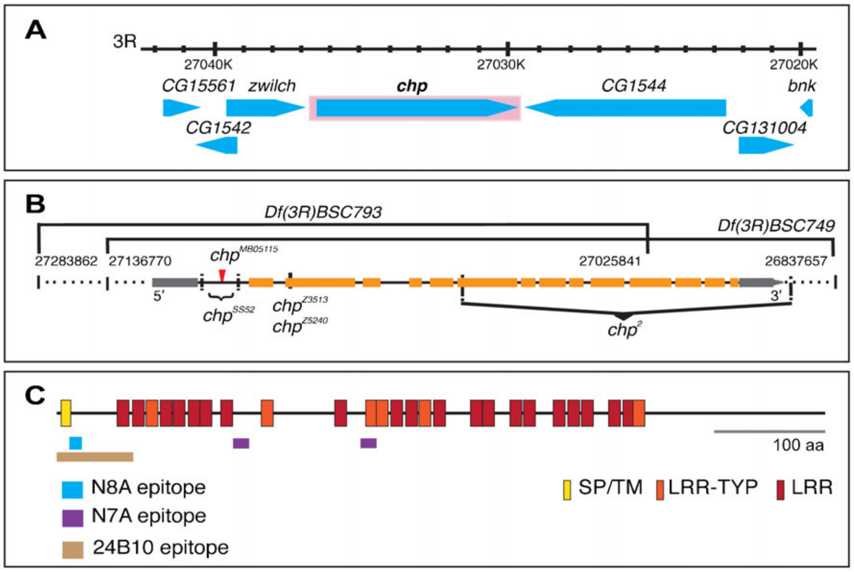

Fig. S3. Genomic organization and encoded putative protein domains of the *chp* gene. (A) Physical map of the *chp* locus on the right arm of the 3rd chromosome. Distal is left. (B) Mapped genetic lesions in the *chp* locus, here projected onto one of the five predicted transcripts, *chp*-RA. Refer to supplementary material Table S1 for details on various *chp* mutant alleles used in this study. (C) SMART annotation for Chp-PA. Localisation of epitopes used to raise Chp antibodies are indicated by blue, magenta and brown bars. Blue: N8A (Yano et al., 2012); magenta: N7A (this study) and brown: 24B10 (Zipursky et al., 1984). SP: signal peptide; TM: trans-membrane domain; LRR-TYP: Leucine Rich Repeat-Typical domain and LRR: Leucine Rich Repeat domain.

Biology Open

Biology Open (2014) 000, 1–10 doi:10.1242/bio.20147310

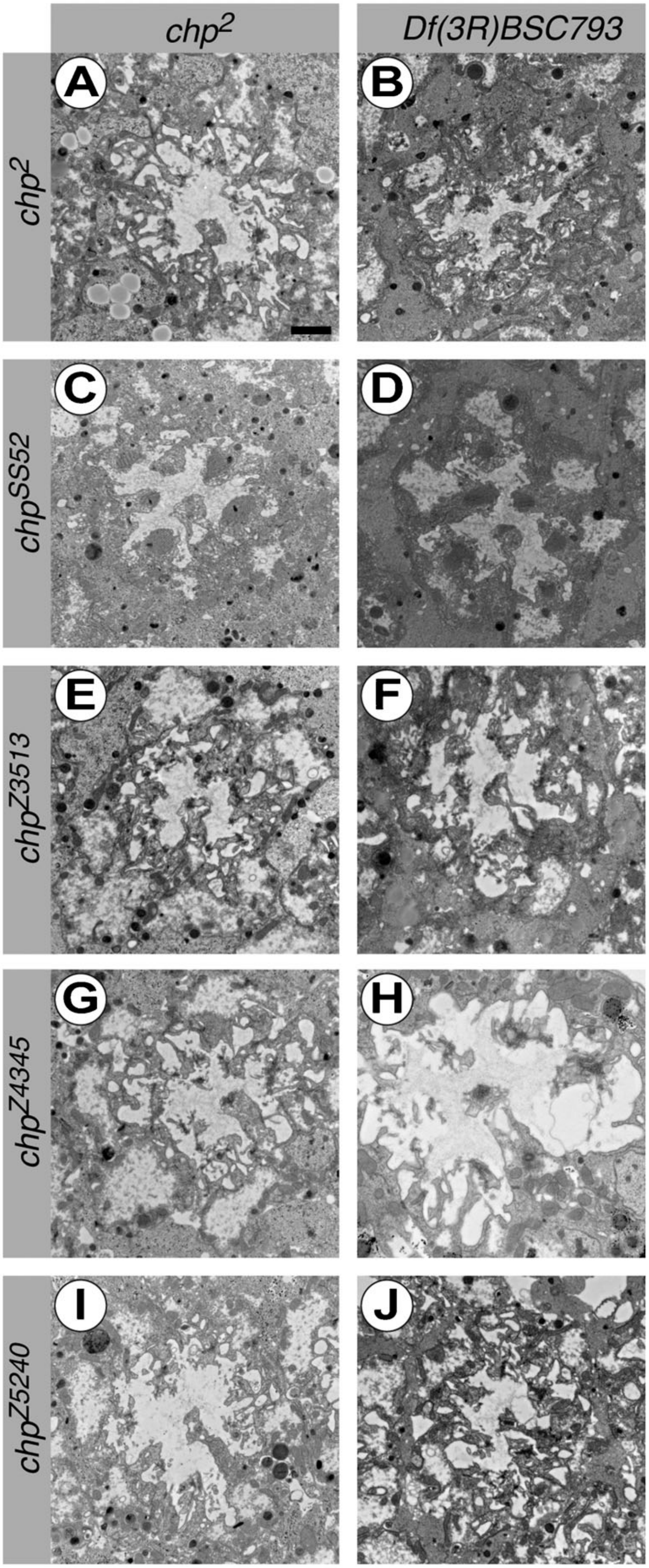

Fig. S4. Phenotype of different *chp* alleles in heteroallelic combinations. Electron micrographs showing tangential distal sections through various *chp* alleles in trans over *chp*^{2} (A,C,E,G,I) and over a genomic deficiency (B,D,F,H,J). Scale bar: 1 µm.

Biology Open (2014) 000, 1–10 doi:10.1242/bio.20147310

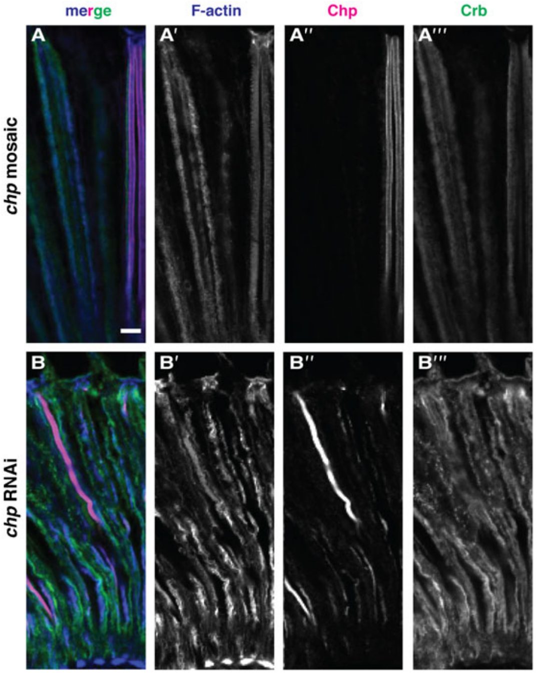

Fig. S5. Chp affects localization of Crumbs in adult PRCs. Confocal images of longitudinal sections through *chp*^{2} *mosaic* (A) and *elav-GAL4>chp*-RNAi (B) ommatidia stained for F-actin (blue), Chp (magenta) and Crb (green). The rudimentary rhabdomeres span the entire retina and show non-uniform F-actin and fuzzy Crb distribution on the apical surface. Scale bar: 5 µm.

Table S1. List of *chp* alleles used

Allele (reference)	Mutagen	Genetic lesion	Phenotype
chp^2 (Van Vactor et al., 1988)	X-ray	Truncation, exon 6 to 3′ (Van Vactor et al., 1988; this study)	Amorph; rudimentary rhabdomeres, no Chp protein detected by immunohistochemistry and western blot
chp^{Z3513} (Sanxaridis and Tsunoda, 2010)	EMS	11 bp deletion in exon 3 (this study)	Hypomorph; Chp misdistributed
chp^{Z5240} (Sanxaridis and Tsunoda, 2010)	EMS	11 bp deletion in exon 3 (this study)	Not determined, homozygous lethal
chp^{Z4345} (Sanxaridis and Tsunoda, 2010)	EMS	No mutation detected within introns and exons (this study)	Hypomorph, Chp misdistributed
chp^{MB05115} (FlyBase; Venken and Bellen, 2007)	Minos-transposon insertion	Minos transposon inserted into the first intron (3R:27035655;27035655, Flybase)	Hypomorph, phenotype stronger in R7
chp^{SS52} (this study)	Minos-transposase	Deletion within the first intron	Hypomorph, Chp protein misdistributed, phenotype stronger in R7 in trans over, deficiency R7 phenotype is stronger than *chp*^{2}, R1–R6
Df(3R)BSC793 (FlyBase; Cook et al., 2012)	FLP-recombinase	Chromosomal deletion, 3R:26837657;27136770	Not determined, homozygous lethal
Df(3R)BSC749 (FlyBase; Cook et al., 2012)	FLP-recombinase	Chromosomal deletion, 3R:27025841;27283862	Not determined, homozygous lethal

chp^{MB05115} is a mutation induced by the insertion of a *minos*-element in the first intron (Flybase). *chp*^{SS52}, which was obtained by transposase-induced imprecise excision of the *minos*-element in *chp*^{MB05115} (see Materials and Methods), is a hypomorphic allele, which severely affects R7 (supplementary material Fig. S3C,D). Its phenotype seems to be slightly stronger than that of the original insertion line. *chp*^{Z3513}, *chp*^{Z5240} and *chp*^{Z4345} are EMS (ethylmethansulfonate)-induced alleles from the Zuker collection (Koundakjian et al., 2004), which have been identified as *chp* alleles (Sanxaridis and Tsunoda, 2010). *chp*^{Z3513} and *chp*^{Z5240} carry the same 11 bp deletion in exon 3 (supplementary material Fig. S2B), which results in a frameshift and a premature stop codon after another 80 unrelated amino acids. No sequence change could be detected in the intron and exon regions of *chp*^{Z4345}. The phenotype of these three alleles in trans over *chp*^{2} or a genomic deletion is as strong as that of *chp*^{2}, suggesting that they are complete loss of function alleles (supplementary material Fig. S3).

Biology Open
